# Morphological and molecular characterization of Brazilian populations of *Diatraea saccharalis* (Fabricius, 1794) (Lepidoptera: Crambidae) and the evolutionary relationship among species of *Diatraea* Guilding

**DOI:** 10.1371/journal.pone.0186266

**Published:** 2017-11-16

**Authors:** Fabricio J. B. Francischini, Jaqueline Bueno de Campos, Alessandro Alves-Pereira, João Paulo Gomes Viana, Christopher C. Grinter, Steven J. Clough, Maria I. Zucchi

**Affiliations:** 1 Department of Genetics and Molecular Biology, Institute of Biology, Universidade de Campinas, Campinas, São Paulo, Brazil; 2 Department of Entomology, California Academy of Sciences, San Francisco, California, United States of America; 3 Department of Crop Science, USDA-ARS / University of Illinois, Urbana, United States of America; 4 Agência Paulista de Tecnologia dos Agronegócios (APTA), Piracicaba, São Paulo, Brazil; Chinese Academy of Agricultural Sciences, CHINA

## Abstract

The sugarcane borer or corn stalk borer, *Diatraea* Guilding is polyphagous insect pest of many important crops such as corn, sorghum and sugarcane. Losses arising from the attack of *Diatraea* species have been a serious problem, which may cause loss in sugarcane production around 0.25% in sugar, 0.20% in alcohol and 0.77% of body weight for every 1% infestation and up to 21% in corn production fields. In Brazil, the most commonly reported species are *Diatraea saccharalis* (Fabricius, 1794) and *Diatraea impersonatella* (Walker, 1863) (= *D*. *flavipennella*). However, multiple other species of *Diatraea* have been identified in Brazil according to the literature. Currently, little information exists on the presence of the other species causing injury to sugarcane and corn. The objectives of this study were to improve the accuracy of species assignment, evaluate the population genetic structure, and address many of the outstanding questions of systematics and evolution of Brazilian populations of *D*. *saccharalis*. To address these main questions, classical taxonomic methods were used, focused on morphological characterization of the reproductive organs, especially the male genitalia. In addition, genetic studies were performed using simple sequence repeats (SSR) and a fragment of cytochrome C oxidase subunit I (COI) gene. The data and findings from this research will contribute to the understanding of evolutionary aspects of insect pests in order to develop more effective and sustainable population management practices.

## Introduction

*Diatraea* Guilding is a genus that is composed of a significant number of species, which contains some of the most important lepidopteran pests of crops, including corn, sorghum, and sugarcane [1, 2, 3, 4]. The species of this genus are restricted to the New World, being widely distributed throughout South America, Central America, the Caribbean, and the southern United States [5, 6, 7, 8]. Currently, in Brazil, all *Diatraea* species are popularly known as sugarcane or corn stalk borers. Because of the lack of distinction between species, borers discovered in agricultural fields are known as *Diatraea saccharalis* (Fabricius, 1794). Based on this principle, *D*. *saccharalis* has been reported to be distributed in sugarcane belts across Brazil [9], whereas *Diatraea impersonatella* (Walker, 1863) another important pest, has been reported only in some states, such as Espírito Santo, Rio de Janeiro, Minas Gerais, and in all the Northeastern states [10, 11, 12]. These two species of *Diatraea* can be morphologically distinguished in the larvae phase. *D*. *saccharalis* larvae has a reddish brown head or dark. The body often dirty white with the pinacula golden brown being that the D2 on A1-7 tend to be on oval to rectangular. The anal shield usually pale [13]. Larvae of *D*. *impersonatella* have a head capsule that is yellow or brownish. The body is cream or yellowish with mesothoracic and metathoracic extra pinacula oval with a median indentation. In addition, the anal crochets in a short arc. The mandible with five teeth [10, 11, 13].

In spite of these differences, farmers, students, and researchers often fail to separate these species correctly. The confusion is likely due to the variability of these characteristics between individuals according to the age of the larvae and environmental conditions [14, 15, 16, 17] and the lack of comparative morphological studies across larvae and pupae to confirm the species level.

The incorrect identification may cause problems for the control of sugarcane borers, given that all actions are targeted towards *D*. *saccharalis*. Chemical control of populations is often not effective given the endophagous feeding habits of the insect and the constant availability of host plants in the field throughout the year [18]. Appropriate biocontrol methods, in many cases, have a high degree of host specificity [12, 19].

Historically there has been very little systematic research conducted on which species are infecting Brazilian corn and sugarcane fields. We aim to develop a comprehensive understanding of the relationships and diversity of *Diatraea* species in support of Brazilian agriculture. Other species of *Diatraea* have also been reported in the Brazilian territory. The first report of *Diatraea* in Brazil was by Dyar [20], who described seven species. The same researcher, in 1927, reported 12 *Diatraea* species in Brazil [1]. Box [5] revised the genus and increased the number of species in the Americas to 48, with 16 found in Brazil. Almeida and Souza [21] reported the occurrence of 10 species attacking sugarcane in Brazil, however, Box [22] narrowed that list down to three: *D*. *saccharalis*, *Diatraea albicrinella* (Box), and *D*. *impersonatella*. Cruz [23] published the last informative survey on the presence of *Diatraea* species within Brazil, reported 10 species in the country, and proposed that *D*. *saccharalis* was the sole pest in sugarcane stalks.

Box [5] and Bleszynski [6] performed very comprehensive studies on the genus *Diatraea* based on the morphology of the male and female genitalia. Expanding this study, Solis and Metz [24], provided keys and illustrations of the male and female genitalia (including many primary types) for all known *Diatraea* species. Based on the variability of South American species, they synonymized the name *Diatraea flavipennella* (Box, 1931) with *D*. *impersonatella*, and we follow the change herein.

The collection and observation of adult Lepidoptera is often preferred given the relative ease of identification. The internal morphological characterization of the anatomy of the reproductive organs, especially the male genitalia, has provided excellent results because they have important and conserved characteristics within species [25, 26, 27]. The genitalia are complex, heavily sclerotized, and provide the basis for discrimination of many species and families [28]. The arrangement of the genitalia is important in courtship and mating feasibility, and can prevent attempted interspecific crosses and hybridization. The Lepidoptera male and female genitalia are adapted one to each other, like a lock and key mechanism [29, 30].

In addition to morphological characterization, molecular analyses may be used to increase the knowledge of a particular group of organisms [31]. Several molecular techniques have been widely used to delimit species, understanding the levels of population diversity, conducting phylogenetic analyses, and estimating gene flow among insect populations [32, 33, 34, 35]. For the identification of species, the most commonly used genetic marker is the polymorphism of sequence of cytochrome C oxidase subunit I (COI) gene. This relatively conserved gene has been useful for alpha level taxonomy because it is generally haploid, lacks introns, and has limited recombination [36, 37, 38, 39, 40]. Another useful molecular tool to study the diversity at the intraspecific level, are the microsatellite markers (SSRs). SSR markers are highly polymorphic and abundant in eukaryotic genomes, and they provide easy and reliable co-dominant genotyping [41, 42, 43].

Very little research on the genetic diversity and population structure of *Diatraea* species have been published in recent years. Some examples showed that Brazilian populations of *D*. *saccharalis* have a high level of polymorphism and genetic structure within the crop production regions [44, 45]. Studies using molecular markers have also shown the possible occurrence of more species than the single currently designated *D*. *saccharalis* [15, 46]. Pashley et al. [15] compared specimens collected in different countries and hosts, and found a cluster with populations from Louisiana and Mexico, and another cluster with Brazilian populations. Joyce et al. [46] studied populations of *D*. *saccharalis* collected in the southern United States and identified two genetically distinct clusters using amplified fragment length polymorphism (AFLP) and COI sequencing. These authors suggested that Florida’s *D*. *saccharalis* population could represent a distinct species. However, other studies suggest samples in South America form a separate cluster from those in Central America and the Southern United Stated of America [7, 47].

Discrepancies among studies may be associated with collection sites, individuals that were collected in light trap or in a host, development stage (adult or larvae) used for DNA analyses, and the lack of precision with species level identification. We addressed these disagreements with a sequence of methods that valid each other. This study aimed to fill a gap, building a comprehensive study of *Diatraea* specimens collected in sugarcane and corn plants in the same production regions of Brazil. Our objectives were (a) to improve the accuracy of species identification through morphological characterization and with the polymorphism of sequence of cytochrome C oxidase subunit I (COI) gene (b) to establish a systematics and evolutionary comparison with *Diatraea* species and (c) to evaluate many of the outstanding questions about population structure with microsatellite (SSRs) fine-scale gene characterizations. In this study, we used these methods to assess the genetic variability of populations collected in the main crop production regions in Brazil. Furthermore, we used DNA barcodes of several species of *Diatraea* to evaluate the evolutionary relationships within this genus. The findings of this study may help to refine the understanding of evolutionary aspects of insect pests in order to develop more effective and sustainable population management practices. In addition, we adopt the use of *D*. *impersonatella* [48] instead of *D*. *flavipennella* [5] as proposed by Solis and Metz [24].

## Materials and methods

### Insect collections

During the Brazilian crop seasons of 2011–2012 and 2012–2013, 95 specimens of *Diatraea* were collected in the main corn and sugarcane production regions of Brazil. The larvae were collected in equidistant points inside the corn or sugarcane fields. In this study, we defined the term "population” as the city where collection was performed, and the host from where the insect was isolated. Thus, each population was defined as Piracicaba_Sugarcane, Piracicaba_Corn, Jaboticabal_Sugarcane, Morrinhos_Sugarcane, Morrinhos_Corn and Maceio_Sugarcane (Table 1).

**Table 1:** Populations collected and their geographic locations.

Species	Collection Location_Host	Latitude	Longitude	Number of Individuals	Female	Male
*Diatraea impersonatella*	Maceio_Sugarcane	-09° 39' 57''	-35° 44' 07''	13	11	2
*Diatraea saccharalis*	Jaboticabal_Sugarcane	-21° 15' 17''	-48° 19' 20'	24	11	13
	Morrinhos_Corn	-17° 43' 52''	-49° 05' 58''	8	3	5
	Morrinhos_Sugarcane	-17° 43' 52''	-49° 05' 58''	2	1	1
	Piracicaba_Corn	-22° 43' 31''	-47° 38' 57''	24	13	11
	Piracicaba_Sugarcane	-22° 51' 31''	-47° 77' 35''	24	9	15

Corn and sugarcane stalks showing the typical symptoms of attack by *Diatraea* borer were cut with the aid of a saw. The larvae found in each damaged plant were transferred to the laboratory, moved to Petri dishes containing artificial diet, identified, and kept separate. Each larva was assessed for the presence or absence of parasites. Larvae that were infected by parasites or other diseases were discarded. The specimens that passed visual screening were placed individually on Petri dishes with artificial diet and maintained at 27 ± 1°C; 70% U.R. and photoperiod of 12 hours until they pupated. Then each individual pupa was transferred to cylindrical cages of 40 cm x 30 cm. The pupae were kept at 20 ± 1°C with a 12-hour photoperiod until adults emerged. Moth emergence occurred within these cylindrical cages to allow the complete metamorphosis and for the moth to properly inflate their wings. All moths were transferred to individual micro centrifuge tubes and stored at– 80°C.

The collections were carried out on private lands (with the permission of their owners), and no specific permits were required for these locations/activities because it did not involve endangered or protected species.

### Morphological characters of the male and female genitalia

All moths that emerged were dissected and identified based on the morphological characteristics of the genitalia according to Robinson [49]. The technique consists of removing the abdomen using curved forceps that are gently pressed on the venter of the caudal end. Abdomens were placed in a 10% potassium hydroxide solution (KOH) and heated to boiling for 2–3 minutes. After this process, the genitalia were transferred to a Petri dish for cleaning in 50% ethanol. Scales were brushed from the abdomen and the genitalia was dissected with the aid of forceps and fine paint brushes under a binocular stereo microscope. Male specimens were dissected by gripping the anterior end of the abdomen while the sclerotized genitalia were gently pulled out of the posterior end. Females were dissected by cutting between abdominal segments VI and VII with the aid of forceps and iris scissors. The extracted genitalia were transferred to small polyethylene tubes (60 mm long by 5 mm diameter) containing glycerin or 85% lactic acid. Each tube contained an individual abdomen with a unique identifier to carefully maintain the association of the genitalia with the specimen. The genitalia of each species were deposited as vouchers in the collection of the Illinois Natural History Survey at the Prairie Research Institute of the University of Illinois under the catalog codes INHS_814844 and INHS_814845. Photographs of representative dissections were taken with a Canon EOS 5D Mark II body and a MPE 65 mm 1x—5x magnifying lens. The z-stacking camera setup was built by Visionary Digital and housed within the entomology collections at the Illinois Natural History Survey at the Prairie Research Institute of the University of Illinois. Photographs were first edited in Adobe Lightroom and then combined with the software package Zerene Stacker. The comparative analysis of male and female genitalia was performed following the description of the *Diatraea* morphology provided by Bleszynski [6] and Solis & Metz [24].

### DNA extraction

The same specimens that were dissected were also used for DNA extractions. The male and female DNA was extracted from the thoracic tissues following the CTAB protocol described by Doyle and Doyle [50] with slight modifications. The integrity and quantity of DNA were evaluated in 0.8% agarose gels with 1x TAE buffer (TRIS, acetic acid, EDTA, pH 8.0). The amount of DNA present in each sample was estimated by comparison with known concentrations and graded standard DNA (λ phage). The gels were stained with an ethidium bromide bath (0.5 mg mL-1) and the DNA bands were visualized under UV light.

### Analysis of the mitochondrial gene cytochrome C oxidase subunit I (COI)

#### Amplification, sequencing and alignment

A fragment of the COI mitochondrial gene was amplified by polymerase chain reaction (PCR) with the primers LCO 1490 (F) (5’—GGT CAA CAA ATC ATA AAG ATA TTG G– 3’) and HCO 2198 (R) (5’—TAA ACT TCA GGG TGA CCA AAA AAT CA– 3’) [51]. The sequencing reactions were performed with the corresponding amplifying primers from both directions using a BigDye Terminator Cycle Sequencing Kit v.2.0 (Applied Biosystems, USA) and the sequences obtained were processed by the 3730 / 3730xl Data Collection Software v3.0 (Applied Biosystems).

Multiple alignment of the sequences (Data available as Supporting Information: S1 Dataset) was done using the ClustalX Software [52] with manual correction using Chromas 2.0 (http://www.technelysium.com.au/chromas.html).

Estimates of haplotype diversity were obtained with DnaSP5 [53]. The distribution of genetic diversity between and within populations and species were estimated by analysis of molecular variance (AMOVA) with Arlequin 3.5 [54]. Cluster analysis was performed with the neighbor joining [55] method using the MEGA4 [56].

The COI haplotypes found for *D*. *saccharalis* and *D*. *impersonatella* were also aligned with COI sequences from other *Diatraea* and related species available in GenBank (COI accession numbers: JQ888353, JQ888360, JQ888366, KJ657593, KM288999, KM289005, KP259615, KR070995, KR070998 and KR070999).

### Analysis with nuclear microsatellite loci

#### Microsatellite genotyping

The microsatellite loci used in the study were provided by the Laboratory of Conservation Genetics and Genomics, Agribusiness Technological Development of São Paulo, Brazil and were developed by Pavinato [57]. Details and characteristics of the microsatellites are shown in S1 Table. Microsatellite amplification conditions and gel separation were performed according to Pavinato [57].

#### Microsatellite data analysis

All individuals from *Diatraea* populations were genotyped with at least 10 highly polymorphic loci (Data available as Supporting Information: S2 Dataset). Genetic diversity and F statistics were estimated under a random model, in which the sampled populations were considered representative of the species and with a common evolutionary history. Allele frequencies, the number of alleles per locus (A), the observed heterozygosity (Ho) and expected (He) and Wright's F statistics (F_IS_, F_ST_ and F_IT_), assuming random model, were estimated using the *hierfstat* package [58]. Cluster analysis were based on the construction of dendrograms using Nei’s genetic distance [59] and the UPGMA method, in the *poppr* package [60]. The stability of the clusters were tested, through 1,000 bootstraps resamples, also using the *poppr* package [60]. We applied the non-model based approach DAPC through the package adegenet [61].

## Results and discussion

### Morphological description of *Diatraea* genitalia

For all 95 individuals collected and reared from sugarcane or corn we performed morphological analysis of the internal genitalia. Specimens were sorted by sex, totaling 47 males and 48 females (Table 1) following keys by Bleszynki [6] and Solis and Metz [24]. We observed that 82 individuals showed little variation in the reproductive organs, independent of the site/city and host in which they were collected. These individuals were identified as *D*. *saccharalis* (Table 1). In addition, we observed that 13 individuals collected at Maceio, in Alagoas State, differed from the other 82 individuals. Using the above mentioned identification guides, we classified this group as *D*. *impersonatella*.

The results clearly demonstrate that all specimens of *Diatraea* collected randomly in sugarcane and corn plants were members of either *D*. *saccharalis* or *D*. *impersonatella*. While other *Diatraea* species are known to exist in Brazil, none was found in these sampling efforts. Interestingly, *D*. *impersonatella* was completely absent from fields of corn and sugarcane in São Paulo and Goiás state. However, *D*. *impersonatella* was the only species reared in the Northeast region of Brazil. Freitas [11] also noticed that *D*. *impersonatella* (89.80%) showed preponderance over *D*. *saccharalis* (10.20%) in some areas of the state of Alagoas.

#### Comparative morphology of the male genitalia

**Fig 1:**
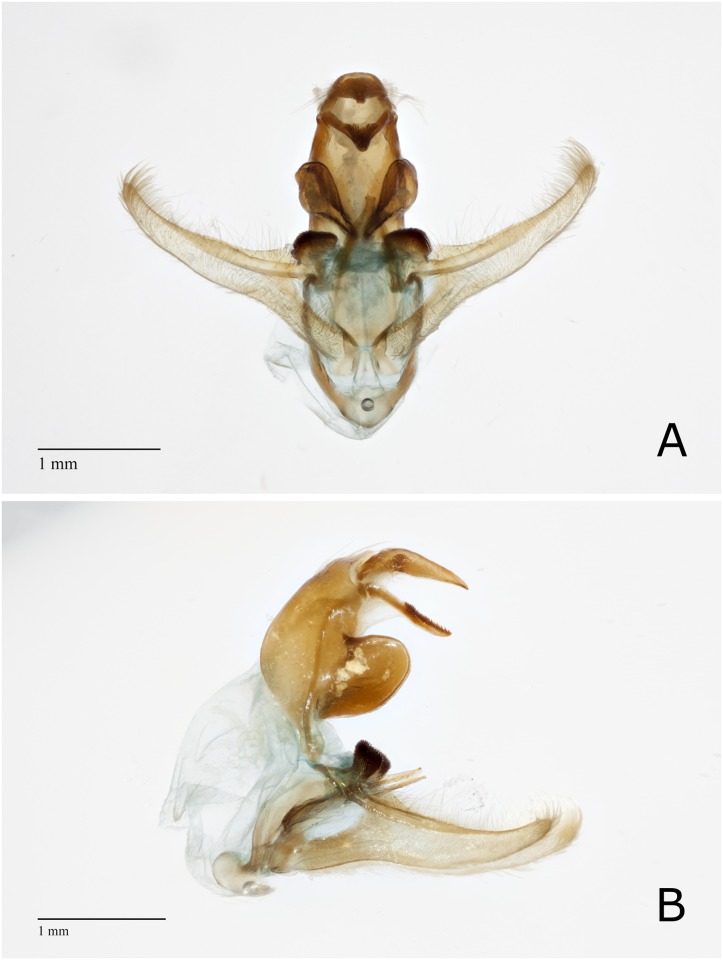
*Diatraea saccharalis* male reproductive structures. (A) Posterior view of *D*. *saccharalis* male reproductive structures. (B) Lateral view of *D*. *saccharalis* male reproductive structures.

Forty-five male specimens were identified as *D*. *saccharalis* according to keys in Bleszynki [6], Goyes [62], and Solis and Metz [24]. The following important morphological characteristics are noted. The *vinculum* was smooth, U-shaped, and broadly rounded anteriorly. The *uncus* narrowed into a beak-like apex, and the *gnathos* were smooth except for approximately half of the dorsal surface that was densely covered with short teeth. The lateral lobe of the *tegumen* was rounded, as long as wide, and square in appearance. The basal costal lobe was present with the vertex slightly flattened. The apex of *juxta* arms were with a single point or rounded with a small, subapical tooth, but never bidentate (Fig 1A and 1B).

**Fig 2:**
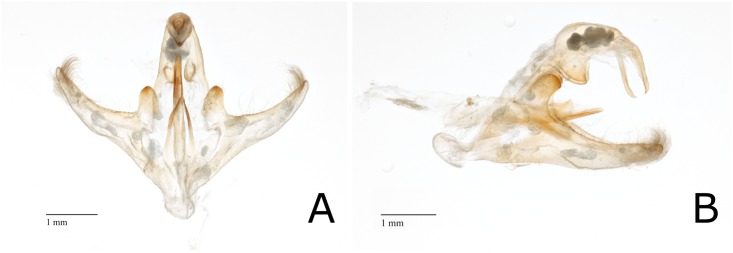
*Diatraea impersonatella* male reproductive structures. (A) Posterior view of *D*. *impersonatella* male reproductive structures. (B) Lateral view of *D*. *impersonatella* male reproductive structures.

*D*. *impersonatella* is similar in appearance to *D*. *saccharalis* in general structure. The *uncus* and *gnathos* were beak-like, valva narrow, and the basal costal lobe pronounced as in *D*. *saccharalis*. Likewise, the lateral arms of *juxta* were slender and pointed. However, the basal costal lobe on the *valva* was elongated and narrow, less dentate, and not as darkened as the lobe of *D*. *saccharalis*. The lateral lobes of the *tegumen* were reduced and triangular in appearance (Fig 2A and 2B). Males of *D*. *saccharalis* and *D*. *impersonatella* can easily be separated from each other, as well as from other members of the genus.

#### Comparative morphology of the female genitalia

**Fig 3:**
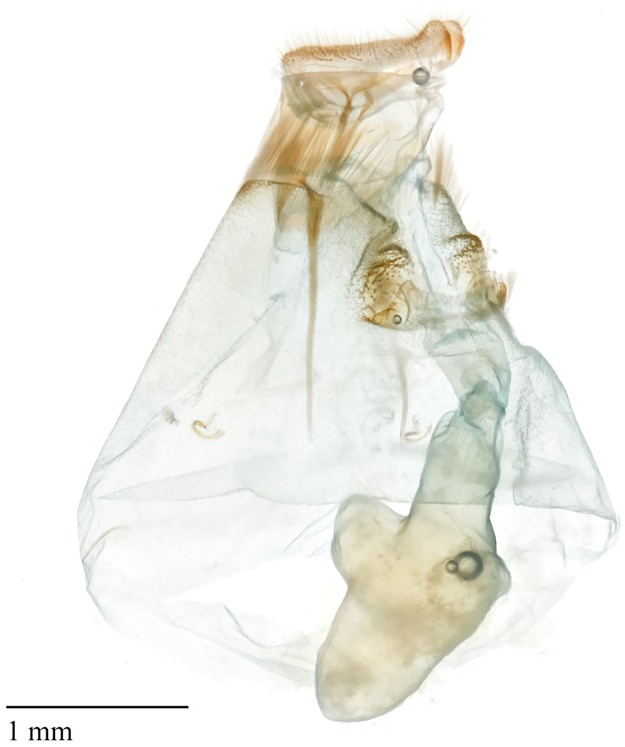
Female reproductive structures of *D*. *saccharalis*.

**Fig 4:**
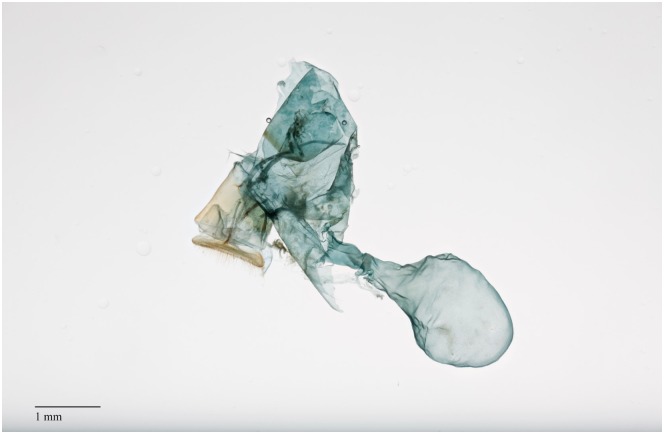
Female reproductive structures of *D*. *impersonatella*.

Females of both *D*. *saccharalis* and *D*. *impersonatella* have *papillae anales* separated anteroventrally, parallel and flattened, with longer setae of the outer margin (Figs 3 and 4). Ventral lobes of the *anales* were slightly swollen. The anterior *apophysis* nearly twice as long as the posterior *apophysis*, slightly curved, and marginally tapering anteriorially. The sternite VIII were with broad, transverse, indentations obstructing the *ostium bursae*. The *corpus bursae* were membranous, lacking any crenulations, and the signa was absent. Moths of *D*. *saccharalis* had an anterodorsal lobe off the *corpus bursae*, although it varied in size and shape. Careful dissection of the female was required to ascertain the true shape of the *bursae*, which can be easily ruptured or crushed. Posterior projections of the lamella *antevaginalis* were irregular and triangular, wrinkled and densley setose. *Ductus seminalis* originated at the posterior end of the *corpus bursae*. In specimens of *D*. *saccharalis* from this study, the anteroventral swelling of the *papillae anales* were more pronounced than in *D*. *impersonatella*. Females of these species cannot reliably be separated by genitalia, and this study lacked sufficient numbers of specimens to ascertain regional variation. Solis and Metz [24] further described variation across the range of the *D*. *impersonatella* group, and the problems with determining species based on female genitalia.

### Analysis of COI mitochondrial gene

**Fig 5:**
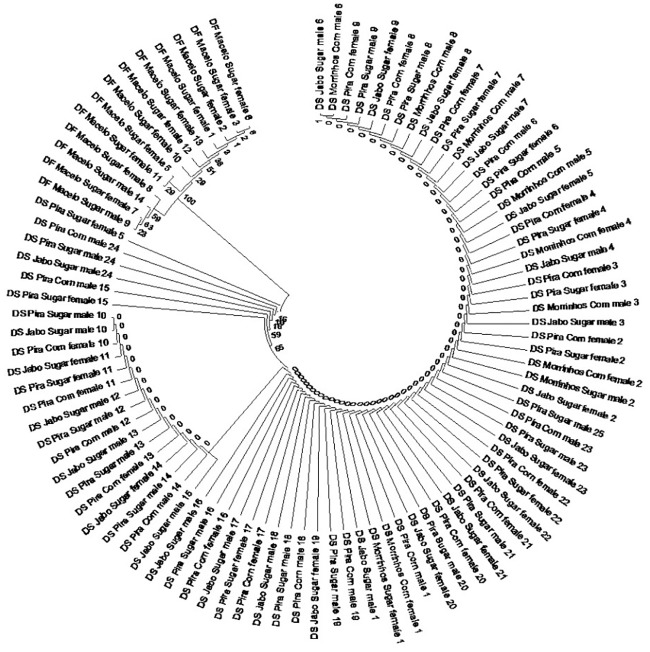
Relationships among individuals of *D*. *saccharalis* and *D*. *impersonatella* from the COI gene.

In addition to classification by taxonomy, we also classified the 95 individuals collected based on DNA polymorphism of sequence of cytochrome C oxidase subunit I (COI) gene. The COI sequence polymorphisms allowed the estimation of the genetic relationships among individuals. Aligned sequences of DNA consisted of 666 base pairs for the COI mitochondrial gene for both species. The average frequency of A, C, G and T was 37.8%, 15%, 16.1% and 31.1%, respectively. The strong AT bias (68.9%) is typical of insect mitochondrial genomes [38, 63]. Neighbor-joining clustering of the COI sequences using Jukes & Cantor distance [64] produced two well-defined groups that perfectly matched the taxonomic findings: one with the 82 individuals identified as *D*. *saccharalis*, while the other one with the 13 individuals of *D*. *impersonatella* separated by a node with 100% of consistency (Fig 5). This result suggests that *D*. *saccharalis* and *D*. *impersonatella* clearly differ from each other. This grouping pattern was expected, because the COI is an efficient method for separation and confirmation of species [40, 65].

**Fig 6:**
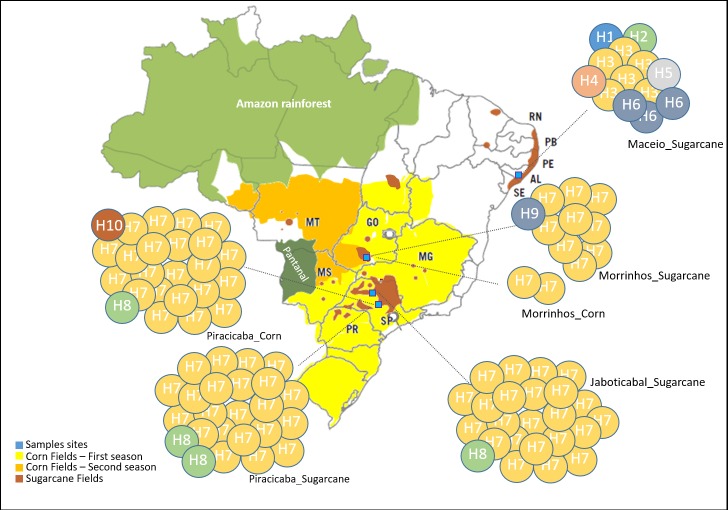
Geographic distributions of COI haplotypes of *D*. *saccharalis* and *D*. *impersonatella*. Each colored circle represents the haplotypes identified in a given population. The number within circles denote the COI haplotypes identified in each population. The descriptions refer to the sampled locations and crops.

A total of 10 haplotypes were obtained from the COI sequencing, showing consistency with previous results (Table 2). Six of the haplotypes were present in *D*. *impersonatella* specimens and four were present in *D*. *saccharalis* specimens (Fig 6). The *D*. *impersonatella* population had the highest haplotype diversity (Table 2 and Fig 6).

**Table 2:** Genetic characterization of populations based on mitochondrial COI barcode sequence analysis.

Species	Number of Individuals	Number of Haplotypes	Haplotype Diversity	Nucleotide Diversity	Tajima's D test (p value)	Fu's Fs test (p value)
*Diatraea impersonatella*	13	6	0.769	0.00316	-0.25752 (= 0.437)	-1.00562 (= 0.246)
*Diatraea saccharalis*	82	4	0.14	0.00032	-1.73486 (= 0.008)	- 2.52261 (= 0.0016)

All individuals of this species were collected in a unique location, and had sugarcane as their host. Haplotypes H3 and H6 were the most frequently found in this species (6/13) and (3/13), respectively. Four haplotypes (H1, H2, H4 and H5) occurred in only one individual. In addition, *D*. *impersonatella* showed high haplotype diversity (0.769) and low nucleotide diversity (0.00316) (Table 2) indicating only a small difference between haplotypes. Although haplotype diversity was high, low nucleotide diversity values indicate the existence of few segregating sites across different haplotypes. The combination of high haplotype diversity and low nucleotide diversity, as observed in our data, may be a signature of a rapid demographic expansion from a small effective population size [65]. Recently, there has been an inversion in the prevalence of the *Diatraea* species that attack sugarcane in Alagoas state. During the 1970s, *D*. *saccharalis* was the predominant species [66]. Currently, Freitas et al. [11] observed 10.20% of *D*. *saccharalis* and 89.80% of *D*. *impersonatella* in Alagoas, indicating the recent expansion of species in this region.

*D*. *saccharalis* haplotype diversity and nucleotide diversity were 0.14 and 0.00032, respectively (Table 2), and the population genetic diversity ranged from 0.083 to 0.2 and nucleotide diversity ranged from 0.0003 to 0.00042. In terms of population differentiation, the spatial distribution of haplotypes demonstrates that the *D*. *saccharalis* populations show relatively little divergence, and shared the most common haplotype, H7 (78/82) that was represented in all collection sites. The second most common haplotype was H8 (4/82), being that one individual was collected in Jaboticabal in the sugarcane crop and three in Piracicaba, one was from corn and two from sugarcane. The H9 and H10 haplotypes were represented by unique specimens, and both were collected in corn fields from Morrinhos and Piracicaba. Low genetic variation in the COI sequences was detected in *D*. *saccharalis*. The main production regions where the insects were collected and where they shared the same haplotypes, were located within the approximately 200 km between Jaboticabal and Piracicaba, the 500 km between Jaboticabal to Morrinhos, and the 650 km between Piracicaba to Morrinhos. Collection sites located in the Piracicaba region had a distance of approximately 30 km between each location, and shared the same haplotypes in different hosts. In Morrinhos city the specimens were collected on the same farm, but from different hosts. Thus, the spatial distribution of haplotypes revealed no major groupings of *D*. *saccharalis* haplotypes according to either host plant or geographical location. Each haplotype, when present in two or more individuals, had a wide geographic distribution.

When genetic structure has been influenced by rapid range expansion, the Tajima’s D value is expected to be negative, indicating an excess of rare nucleotide variants compared to the expected under a neutral model of evolution [67]. In this study, the Tajima’s D values were negative for both species, -1.734 (p-value < 0.01) for *D*. *saccharalis*, and -0.25752 (p-value = 0.437) for *D*. *impersonatella*. These results show that the mutations found in the *D*. *saccharalis* sequences probably occurred due a genetic drift, not selective pressure. While for *D*. *impersonatella*, the negative Tajima’s D was not statistically significant, and therefore the hypothesis of neutral evolution was rejected for this species. The results of Fu's FS test, which is based on the distribution of haplotypes, also had negative values for all *D*. *saccharalis* populations, confirming an excess of rare haplotypes over what would be expected under neutrality. Just as for Tajima’s D, Fu’s FS was not significant for *D*. *impersonatella*.

An analysis of molecular variance (AMOVA) was performed to verify how the genetic variability was distributed among and within the two species collected. Consistent with the other analyses presented here, the AMOVA also shows the separation of the samples into two species of *Diatraea*. The AMOVA results showed a high percentage of variation between species 99.21% (F_ST_ = 0.99). Moreover, low genetic variations were observed among populations within species and within species, -0.02% and 0.81%, respectively (Table 3). An AMOVA with only *D*. *saccharalis* samples revealed that the largest percentage of variation occurred within populations, with 100%, suggesting that the variation was distributed randomly between sites (S2 Table).

**Table 3:** Hierarchical analysis of molecular variance (AMOVA) for population genetic structure of *D*. *saccharalis* and *D*. *impersonatella* based on the variation of the mitochondrial COI gene. D.f. = degrees of freedom.

Hierarchical levels	d.f.	Sum of Squares	Variance components	Variance (%)	Fixation Indices	p-value
Three-hierarchical-levels						
Among species	1	639.475	28.48853	99.21	F_ST_ = 0.99215	< 0.001
Among populations within species	2	0.184	-0.00612	-0.02	F_CT_ = -0.02714	0.4531
Within species	91	21.078	0.23163	0.81	F_SC_ = 0.991913	< 0.001
Total	94	660.737	28.71404			

The high-level divergence between *D*. *saccharalis* and *D*. *impersonatella* is also evident by the F_ST_ estimates among their populations. The pairwise F_ST_ estimates between *D*. *saccharalis* populations ranged from -0.33172 to 0.05546. The highest pairwise F_ST_ estimate was observed between populations from different hosts, Jaboticabal_Sugarcane and Morrinhos_Corn (F_ST_ = 0.05546), however this genetic divergence was not significant (S3 Table).

The relationship between these two species of *Diatraea* has always been unclear, and there is little historical knowledge about demographic expansion, especially for *D*. *impersonatella*. Myers [68, 69] conducted a fascinating global search for primitive habitats and original host-plants of *Diatraea* species. He concluded that *D*. *saccharalis* co-evolved with riparian aquatic vegetation, and that the probable center of origin was between the delta region of the Orinoco River, Venezuela, and the lower Amazon River, Brazil. Taking into account that *D*. *flavipennella* is considered a synonym of *D*. *impersonatella* [5, 6, 24] the species was recorded as an original member of a true savannah and riparian vegetation [69]. During the colonization of Amazon basin these two species shared the same host, *Paspalum fasciculatum* (Poaceae), and was often found in association with insect damage in that region. The expansion of *Diatraea* species to other parts of Brazil is unknown. We can speculate that these pests were introduced into Brazil in the 16th century or later as stalks of sugarcane varieties or native plants from the Amazon basin, were transported throughout the region by colonists.

**Fig 7:**
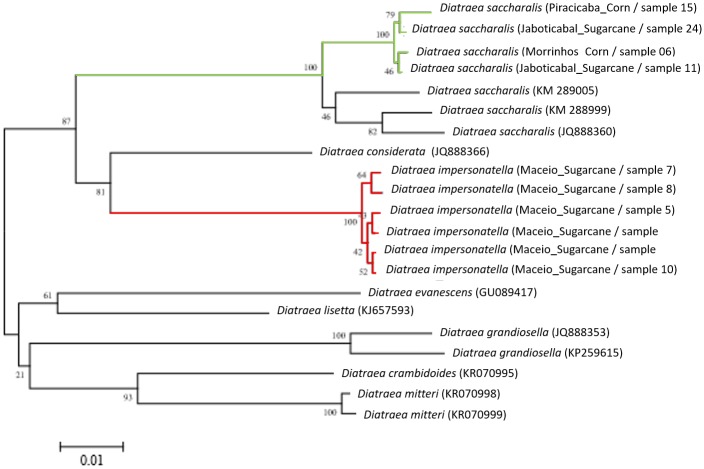
Neighbor joining dendrogram showing the relationships among haplotypes of the COI gene for *Diatraea* species. Individuals collected as part of this study are indicated with colored branches (Green: *D*. *saccharalis* and Red: *D*. *impersonatella*). Individuals showed in black were obtained from GenBank and BOLD databases. Confidence level values were based on 1000 bootstrapping.

Dissimilarities among the species sequences indicate genetic divergence as the result of molecular evolution during the course of time [70]. In analyzes of the COI sequence of these two species, combining with sequence data from other *Diatraea* species (obtained from GenBank and BOLD) showed that these species cluster together. Other clusters contained species originating from other localities and hosts (Fig 7). COI sequences were not available from the public databases for *D*. *impersonatella* or *D*. *flavipennella* to add to that analysis.

Studies with mitochondrial DNA COI and COII sequences of *D*. *saccharalis* populations, including overseas populations, have shown a divergence between individuals and thus a geographic population structure [7, 8, 45, 46]. It is clear in the results of these researchers that there exists a divergence between populations from South America to those collected in the southern USA. These studies, with different molecular markers, were able to identify clusters that can be associated with several introductions of this borer into each country [68, 69, 71, 72]. There have been few studies to determine the population structure of *D*. *saccharalis* in Brazil. Cortes et al. [8] analyzed sequence variation in the barcode region of the COII gene and found low intra-specific variation among samples collected, with 98% of individuals sharing a common haplotype similar to that also found in all samples collected from in São Paulo, Paraná and Pernambuco state. Noteworthy that the most frequent haplotype observed by Cortes [8] had the same sequence previously reported by Lange et al. [7] in a Brazilian population.

In contrast to this observations, Silva-Brandão et al. [45], in a study using COI sequences combined with nad6 sequences, were able to distinguish a high level of genetic structure among samples of *D*. *saccharalis* collected in Brazilian corn and sugarcane fields, obtaining data with high values of F_ST_ and haplotype diversity, but low nucleotide diversity. Interestingly, the same haplotype was found at high frequency (68 of 125 specimens; 54.4%), possibly the consequence of using two molecular makers instead of one. The length of COI and *nad6* sequences used in that study were long, at 1,429 and 497 bases, respectively. The majority of studies involving COI sequences have used a short barcode section of mitochondrial DNA, the first approximate 650 bases of the 5’-end of this gene [40, 73, 74]. Consistent with our work, the most frequent haplotype detected by Silva-Brandão et al. [45] was identical to the most frequent haplotype in the present study. Furthermore, our findings showed no structured genetic variability in populations of *D*. *saccharalis*, and the haplotype diversity was lower than expected, in contrast to published records for this species [7, 45, 46].

Contrasting results may have emerged due to different factors, such as sampling and the methods employed. For example, light traps may attract insects with different hosts, which may increase the diversity sampled. Other important point is the definition of development stage (moth or larvae) used for DNA extraction. Even though the use of larvae is allowed by International Barcode of Life project (iBOL), bacteria present in the larvae body may be a source of contaminant DNA. Thus, sampling of moths is an advantage with respect to the quantity and quality of DNA isolated [75, 76]. Moreover, another important aspect is the precise taxonomic identification to support molecular analyses of the species.

Based on these premises and on examination of almost 100 individuals we can show that low values of haplotype and nucleotide diversity and the higher frequency of the same haplotype is a realistic scenario. The homogeneity observed in COI among widely dispersed and geographically isolated Brazilian populations of *D*. *saccharalis* can be explained as a consequence of low mutation rates and/or stochastic processes that resulted in severe bottleneck of the population sizes, as described by negative values of Tajima’s D. According to Myers [68, 69] the original hosts of *D*. *saccharalis* are aquatic and semi-aquatic grasses in the Orinoco Delta River. Therefore, we may speculate that when sugarcane was introduced in the New World by the sixteenth century, *D*. *saccharalis* migrated, colonized, and adapted to the new hosts and environments. The bottleneck signal observed suggest that during the colonization a relatively small number of individuals founded the new populations [77, 78]. This demographic bottleneck could also be suggested by the genotype and haplotype composition, which indicated a reduced genetic diversity in Brazilian populations of *D*. *saccharalis* [79, 80, 81, 82]. Recently, various studies reported on species that have low mtDNA variation and no population structure [83, 84, 85, 86, 87, 88]. DNA identification will not work unless the variations are much less within a species than between species [89]. According to Hebert et al. [40], the mean interspecific genetic divergence should be at least 10 times higher than the average intraspecific genetic distance in order to define the presence of species complexes. High levels of COI sequence variation within species could complicate efforts to use COI to differentiate between species [65].

In this study, we showed that the analysis of polymorphism of oxidase cytochrome C oxidase subunit I (COI) mitochondrial gene is a powerful and accurate species discriminator for *D*. *saccharalis* and *D*. *impersonatella*. In relation to intraspecific studies within *D*. *saccharalis*, COI analysis did not show genetic structure for populations sampled in this study. The use of combined morphological and molecular approaches, including both mtDNA and a nuclear DNA, is proposed to evaluate relationships that persist uncertain.

### DNA polymorphism analysis with nuclear microsatellite loci

To investigate the genetic variation in populations of *D*. *saccharalis*, we performed microsatellite loci characterization on 80 of the 82 individuals collected (Table 1) with eleven microsatellite loci (S1 Table). Two individuals from Morrinhos_Sugarcane were removed of the study because they did not fit within the criteria for assignment. In the analysis of *D*. *saccharalis* populations using eleven microsatellite loci, we observed that the total number of alleles was 51, ranging from 2 to 7 alleles per locus. The loci Dsc1, Dsc2, Dsc10 and Dsc20 showed the smallest diversity, while the loci Dsc 03, Dsc9, Dsc11 and Dsc20 showed the highest (S4 Table). The average observed heterozygosity per loci was 0.42, ranging from 0.084 to 0.885, while the average expected heterozygosity was 0.49, ranging from 0.122 to 0.632. The average coefficient of inbreeding (intrapopulation fixation index) per loci was 0.14, ranging from -0.413 to 0.472 (S4 Table).

The average number of alleles found was 35.8, ranging from 30 to 40 alleles. The average allelic richness (Ar) per population was 29.83 and ranged from 32.4 to 25.19. The population Piracicaba_Sugarcane showed the highest value of allelic richness. The average values of observed and expected heterozygosity per population were 0.42 and 0.46, respectively. The highest heterozygosity was observed in Morrinhos_Corn (0.44), and the lowest in Jaboticabal_Sugarcane (0.4). For the expected heterozygosity, the highest value was 0.51 for Morrinhos_Corn, and the lowest was 0.39 for Piracicaba_Corn. The expected heterozygosity values were higher than observed heterozygosities for all populations, except for Piracicaba_Corn. The average coefficient of inbreeding was 0.10, ranging from -0.074 to 0.179. The estimate values of F_IS_ was positive for the populations from Jaboticabal_Sugarcane (0.1788), Morrinhos_Corn (0.1429), and Piracicaba_Sugarcane (0.1323), reflecting an excess of homozygotes in these populations, while Piracicaba_Corn was the only population with negative value of F_IS_. These differences among the observed and expected heterozygosities can be attributed to non-random mating among the individuals and inbreeding within populations with positive values of F_IS_. While the negative inbreeding coefficient observed in Piracicaba_Corn suggests that these processes are not occurring in this populations (Table 4).

**Table 4:** Genetic diversity estimates for each population of *D*. *saccharalis* based on eleven microsatellite loci.

Population	n	N_A_	PA	AR	H_E_	H_o_	F_IS_
Jaboticabal_Sugarcane	24	40.0	3	30.93	0.480	0.400	0.179
Morrinhos_Corn	8	34.0	6	30.79	0.510	0.440	0.143
Piracicaba_Sugarcane	24	39.0	3	32.4	0.490	0.420	0.132
Piracicaba_Corn	24	30.0	0	25.19	0.390	0.420	-0.074
**Average**	-	35.8	-	-	0.46	0.42	0.10

n -Number of individuals, N_A_—Number of alleles, PA—Private Alleles, AR—Allelic Richness, H_E_—Expected heterozygosity, H_O_—Observed heterozygosity, F_IS_—coefficient of inbreeding.

We identified 12 private alleles, 50% were found in Morrinhos_Corn, and the Piracicaba_Corn population had no private alleles. The molecular marker, which identified the largest number of private alleles, was Dsc11, with 5 alleles distributed in Morrinhos_Corn, Jaboticabal_Sugarcane and Piracicaba_Sugarcane populations (S5 Table).

**Fig 8:**
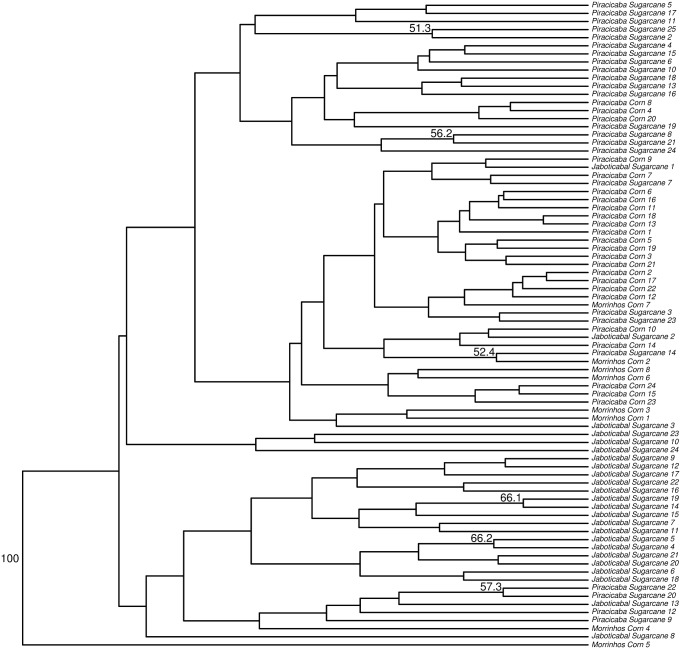
Dendrogram showing genetic relationships among 82 individuals of *D*. *saccharalis*. Neighbor-joining tree based on the pairwise genetic distances between individuals estimated by the logarithm of the proportions of shared alleles, and 1000 bootstrap repetitions.

The values of pairwise F_ST_ indicated genetic differentiation among populations, and ranged from 0.0835 to 0.1812 (S6 Table). We observed that Jaboticabal_Sugarcane and Piracicaba_Corn were the most divergent populations (0.1812), while Morrinhos_Corn and Piracicaba_Corn, with the same host, were the less divergent (0.0835). Using the UPGMA method (unweighted pair-group method with arithmetic mean) [90] a dendrogram based on Nei’s standard distances [91] was generated. In this analysis, we observed the existence of well-defined groups, indicating the existence of genetic structure among hosts (Fig 8), suggesting that the populations of *D*. *saccahralis* collected in sugarcane and corn are not closely genetically related. Additionally, the clusters observed in the dendrogram are also in accordance with the sites of collection, which can be an indication of geographic structure.

**Fig 9:**
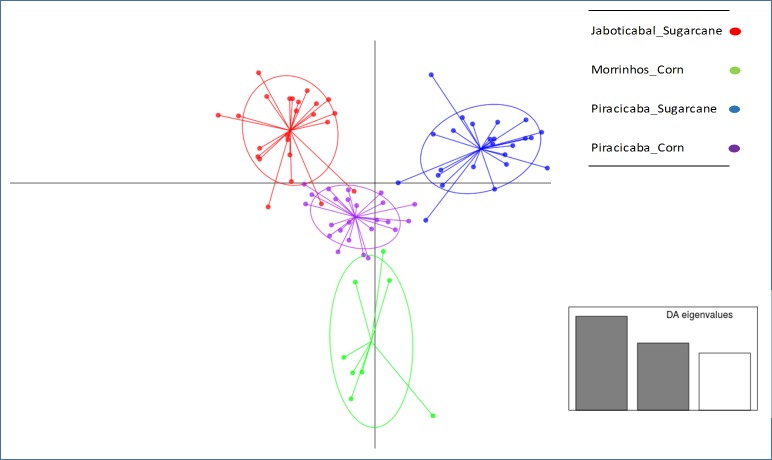
DAPC showing habitat profiling of individuals across four *D*. *saccharalis* populations, using the microsatellite data. Variation represented in x = 42% and in y = 30%.

In the DAPC, 72% of the total genetic variation was captured by components of PCA and these were used as input to capture two DA functions. This analysis separated the samples in four major clusters that correspond to both geographic locations and hosts (Fig 9). Especially in Piracicaba the separation by host can be clearly visualized. The DAPC agreed the pairwise differentiation (F_ST_) estimates among sampling sites.

In Brazil, sugarcane and corn are cultivated in a large diversity of environments, which differ in soil conditions, climate, availability and susceptibility of the variety and management control system. It is characteristic of inconstant environments to exhibit a significant loss and fragmentation of natural ecosystems [92].

Furthermore, these diverse cultivation ecosystems provide habitats for a wide range of pests. Insects demonstrate a high ability of local adaptation and acceleration of the evolutionary process [93, 94]. The fragmentation process can lead to reduced effective insect population sizes and an increase in the mating between relatives. Especially in phytophagous insects, the process of fragmentation drives the reduction of gene flow and increases host plant specialization [95]. Our data show an overall deficit of heterozygotes, and a significant genetic differentiation among populations. According to Avise et al. [36], the dispersion capacity, the geographic barriers and other related process may also affect the population structure of a given species. The agricultural production systems in Brazil may have considerably influenced *D*. *saccharalis* populations. The development of new varieties of sugarcane allowed the cultivation in a system ranging from 12 to 18 months. This sequential production system permits *D*. *saccharalis* to have host plants yearlong and limits the necessity of migration. In corn production systems, the introduction of Bt technology in the mid-1990s created an agricultural system less dependent on insecticides, but selectively eliminates certain species from the insect populations. Additionally, the Bt technology might provide the primary ecological opportunities needed for the first host shift and encourage adaptation to digest novel plant defensive proteins [96, 97, 98, 99, 100]. Another important point to consider is related to the capacity of dispersal of this moth. The dispersal behavior of insects is the major factor that can influence gene flow among their populations [101]. Results of mark–recapture studies suggest that dispersal rates in *D*. *saccharalis* are low, apparently as result of home-range behavior. Over 45% of the adults were recaptured around 50 meters of the release site. Extended dispersal was observed when the moths followed the wind, which increased their dispersal in around 800 meters [102, 103].

Limited flight ability, genotype–environment interactions, and host year-availability affect the relationship of *D*. *saccharalis* with the major hosts and suggest that the current genetic divergence and inbreeding result from limited gene flow and natural barriers. Evidence of fragmentation influence on genetic diversity was found in other studies with *D*. *saccharalis* populations [104, 105]. Pavinato [104] observed divergence and limited gene flow among populations of *D*. *saccharalis* collected in corn and sugarcane, just as the results found in our study. However, Nascimento [105] reported low levels of structuring among populations of *D*. *saccharalis* in sugarcane. This lack of divergence observed between populations collected in sugarcane may be related with the proximity of samples sites, since all populations belonged to the same state in Brazil.

Another important factor for the population divergence observed in our study may involve different selective pressures across environments. In addition, low rates of gene flow between populations increase the likelihood of host-associated genomic differentiation. We found strong evidence of host-associated genetic divergence across the range of *D*. *saccharalis*, such as the DAPC and pairwise F_ST_ results. Pavinato [104] support the hypothesis of ecological divergence between *D*. *sacharalis* populations from corn and sugarcane. Subpopulations of the same host (sugarcane or corn) tend to be more similar to each other [104].

In conclusion, microsatellite loci were polymorphic and highly informative, allowing the study of genetic variability of *D*. *saccharalis* collected in Brazil, which suggested the presence of genetic groups correspondent with their geographic sampling locations. It is possible that these populations have evolved some degree of adaptation to local environmental conditions.

## Conclusions

We conducted a systematic study of *D*. *saccharalis* and *D*. *impersonatella* that included characteristics from genitalia anatomy, mtDNA, and nuclear molecular markers. Our results clarified some outstanding questions about *Diatraea* populations in the Brazilian territory. In this study, we observed, through taxonomic methods and COI sequencing, that *D*. *saccharalis* and *D*. *impersonatella* are the two species responsible for attacking sugarcane and corn in Brazilian crop fields. Sequencing of COI revealed to be an accurate species discriminator for this genus. Microsatellite analyses revealed host-plant preference in populations of *D*. *saccharalis*. Moreover, genetic structure showed little connection among populations. There are preliminary indications that low interactions can relate to the fragmentation process between crop productions regions and may affect gene flow. In summary, we have demonstrated that microsatellite polymorphisms provide a valuable tool for population genetic analysis of *D*. *saccharalis*. Additionally, we strongly recommend the adoption of the name *D*. *impersonatella* as a nomenclatural change from *D*. *flavipennella* as proposed in Solis and Metz [24]. Our findings directly affect the adoption of control actions. The establishment of a functional program that will ensure the design and implementation of sustainable pest management strategies needs to take into account the genetic structure and local characteristics of *Diatraea* populations.

## Supporting information

S1 Table Characteristics of the 11 microsatellite loci from *D*. *saccharalis*. F = forward primer sequences; R = reverse primer sequences; Ta = annealing temperature.
(PDF)

S2 Table Hierarchical analysis of molecular variance (AMOVA) for population genetic structure of *D*. *saccharalis* with a mithocondrial (COI) region marker. (PDF)

S3 Table Estimates of pairwise F_ST_ (lower diagonal) and p-values (upper diagonal) among populations of *D*. *saccharalis* and *D*. *impersonatella*, based on the variation of the mitochondrial COI gene. (PDF)

S4 Table Genetic variability and coefficient of inbreeding for microsatellite loci evaluated in *D*. *saccharalis* populations. (PDF)

S5 Table Nuclear microsatellite private alleles observed in four populations of *D*. *saccharalis*. (PDF)

S6 Table Estimates of pairwise F_ST_ among the *D*. *saccharalis* populations based on the variation of eleven microsatellite loci. (PDF)

S1 Dataset COI data from *Diatraea* populations. (TXT)

S2 Dataset Microsatellite data from *Diatraea* populations genotyped with 10 polymorphic loci. (XLSX)

## References

[1] Dyar HG, Heinrich C. The American moths of the genus Diatraea and allies. Proceedings of the United States National Museum. 1927; 71: 1–48.

[2] Long WH. Hensley SD. Insect pests of sugar cane. Annu. Rev Entomol. 1972; 17:149–176.

[3] Roe RM. A Bibliography of the Sugarcane Borer, Diatraea saccharalis (Fabricius), 1887–1980. USDA, ARS. ARM-S 20, New Orleans; 1981.

[4] Rodrıguez-del-Bosque LA, Smith JW Jr, Browning HW. Bibliography of the neotropical cornstalk borer, *Diatraea lineolata* (Lepidoptera: Pyralidae). The Florida Entomologist. 1988; 71, 176–186.

[5] Box HE. The crambine genera Diatraea and Xanthopherne (Lep. Pyralidae). Bull Entomol Res. 1931; 22: 1–50.

[6] Bleszynski S. The taxonomy of the crambine moth borers of sugar cane pp. 11–59. In Williams JR, Metcalf JR, Mungomery RW, Mather R Pests of sugar cane. Elsevier Publishing Co, NewYork, NY; 1969.

[7] Lange CL, Scott KD, Graham GC, Sallam MNE Allsopp PG. Sugarcane moth borers (Lepidoptera: Noctuidae and Pyraloidea): phylogenetics constructed using COII and 16S mitochondrial partial gene sequences. Bulletin of Entomological Research. 2004; 94, 457–464. 15385065 10.1079/ber2004320

[8] Cortés AMP, Zarbin PHG, Takiya DM, Bento JMS, Guidolin AS, Consoli FL. Geographic variation of sex pheromone and mitochondrial DNA in *Diatraea saccharalis* (Fab., 1794) (Lepidoptera: Crambidae). Journal of Insect Physiology. 2010; 56: 1624–1630. doi: 10.1016/j.jinsphys.2010.06.005 20558173 10.1016/j.jinsphys.2010.06.005

[9] Dinardo-Miranda LL. Pragas In: Dinardo-Miranda, Vasconcelos, de A Landell MG. (Ed.). Cana-de-açúcar. Campinas: Instituto Agronômico p.349–404. 2008.

[10] Mendonça AF. Pragas da cana-de-açúcar. Insetos & Cia, Maceió, 200pp. 1996.

[11] Freitas MR, Fonseca AP, Silva EL, Mendonça AL, Silva CE, Mendonça AL, Nascimento R, Santana AE. The predominance of *Diatraea flavipennella* (Lepidoptera: Crambidae) in sugar cane fields in the states of Alagoas, Brazil. Fla. Entomol. 2006; 89:539–540

[12] Neto MSR. Análise estrutural, aplicação filogenética e barcode do ITS 2 na broca pequena da cana-de-açúcar Diatraea spp (Lepidoptera). Universidade Federal de Pernambuco Centro de Ciências Biológicas Programa de Pós-Graduação em Genética. 2014

[13] Passoa, S. C. Guide to species of Diatraea intercepted or potentially encountered at U.S. ports of entry using morphology and origin, 5 pp. In: Gilligan, T. M. and S. C. Passoa. LepIntercept, An identification resource for intercepted Lepidoptera larvae. Identification Technology Program (ITP), USDA/APHIS/PPQ/S&T, Fort Collins, CO. 2014.

[14] Box HE. New species and records of Diatraea Guild from northern Venezuela (Lepid:Pyral.). Bull Entomol Res. 1951; 42:379–398.

[15] Pashley DP, Hardy TN, Hammond AM, Mihm JA. Genetic evidence for sibling species within the sugarcane borer (Lepidoptera: Pyralidae). Ann Entomol Soc Amer. 1990; 83: 1048–1053.

[16] Riley DR, Solis MA. Keys to immatures of the sugarcane borer and neotropical cornstalk borer from Tamaulipas, México, intercepted on corn in southeastern Texas. Southwestern Entomol. 2005; 30:35–39.

[17] Passoa SC. Morphological guide to known species of Diatraea intercepted at U.S. ports of entry from Mexico, 3 pp. In: Gilligan, T. M. and S. C. Passoa. LepIntercept, An identification resource for intercepted Lepidoptera larvae. Identification Technology Program (ITP), USDA/APHIS/PPQ/S&T, Fort Collins, CO. 2014.

[18] Cirelli KRN, Penteado-Dias AM. Análise da riqueza da fauna de *Braconidae* (Hymenoptera: Ichneumonoidea) em remanescentes naturais da Área de Proteção Ambiental (APA) de Descalvado, SP. Revista Brasileira de Entomologia. 2003; 47:89–98.

[19] Goméz LA, Lastra LA. Los barrenadores de la caña de azúcar. Serie Divulgativa, Cenicaña, 1995.

[20] Dyar HG. The American species of *Diatraea saccharalis* Guigig (lepid., Pyralidae). Ent. News. 1911; 22: 199–207.

[21] Almeida JR, Souza AF. A broca da cana-de-açúcar. Rev. Agric. Piracicaba. 1936; 11: 257–292.

[22] Box HE. The species of Diatraea and allied genera attacking sugarcane. Proc. Int. Soc. Sugarcane Technol. 1960; 10:870–876.

[23] Cruz FZ. Espécies do gênero Diatraea Guilding, 1828 (Lep., CRAMBIDAE) coletadas em Canaviais de algumas regiões do Estado de São Paulo—Brasil. Dissertação (Mestrado em Entomologia)—Escola Superior de Agricultura “Luiz de Queiroz, Universidade de São Paulo, Piracicaba. 1976.

[24] Solis MA, Metz MA. An illustrated guide to the identification of the known species of Diatraea Guilding (Lepidoptera: Crambidae: Crambinae) based on genitalia. ZooKeys. 2016; 565: 73–121. 10.3897/zookeys.565.6797 PMC4820097 27081337

[25] Scudder GGE. Comparative morphology of insect genitalia. Annual Review of Entomology. 1971; 16: 379–406.

[26] Peairs B, Saunders JL. *Diatraea lineolate* y *Diatraea saccharalis*: Una revision em relacion con el maiz. Agronomia Constarricense. 1980; 4(1): 123–135.

[27] Schilthuizen M. Shape matters: The evolution of insect Genitalia. Proceedings of the section Applied and Experimental Entomology of the Netherlands Entomological Society. 2003; 14:9–15.

[28] Powell JA. Lepidoptera In: Encyclopedia of Insects. Resh VH, Carde AT Academic Press; 2009 pp. 1132 ISBN 9780123741448. http://books.google.co.in/books?id=wrMcPwAACAAJ.

[29] Mikkola K. Evidence for lock-and-key mechanisms in the internal genitalia of the Apamea moths (Lepidoptera, Noctuidae). Systematic Entomology. 1992; 17:145–153.

[30] Arnqvist G. The evolution of animal genitalia: distinguishing between hypotheses by single species studies. Biological Journal of the Linnean Society. 1997; 60: 365–379.

[31] Brown B, Emberson RM, Paterson AM. Phylogeny of ‘‘Oxycanus” lineages of hepialid moths from New Zealand inferred from sequence variation in the mtDNA COI and II gene regions. Molecular Phylogenetics and Evolution. 1999; 13: 463–473 doi: 10.1006/mpev.1999.0662 10620404 10.1006/mpev.1999.0662

[32] Sosa-Gómez DR. Intraspecific variation and population structure of the velvetbean caterpillar, *Anticarsia gemmatalis* Hubner 1818 (Insecta: Lepidoptera Noctuidae). Genetics and Molecular Biology. 2004; 27: 378–384.

[33] Martinelli S, Barata RM, Zucchi MI, Silva-Filho MC, Omoto C. Molecular variability of *Spodoptera frugiperda* (Lepidoptera: Nocuidae) populations associated to maize and cotton crops in Brazil. Journal of Economic Entomology. 2006; 99: 519–526. 16686155 10.1603/0022-0493-99.2.519

[34] Martinelli S, Clark Pl, Zucchi MI, Silva-Filho MC, Foster JE, Omoto C. Genetic structure and molecular variability of *Spodoptera frugiperda* (Lepidoptera: Noctuidae) collected in maize and cotton fields in Brazil. Bulletin of Entomological Research. 2007; 97:225–231. doi: 10.1017/S0007485307004944 17524154 10.1017/S0007485307004944

[35] Leite NA, Alves-Pereira A, Correa AS, Zucchi MI, Omoto C. Demographics and Genetic Variability of the New World Bollworm (*Helicoverpa zea*) and the Old World Bollworm (*Helicoverpa armigera*) in Brazil. PLoS One. 2014; 9(11): e113286 doi: 10.1371/journal.pone.0113286 25409452 10.1371/journal.pone.0113286 PMC4237417

[36] Avise JC, Arnold J, Ball RM, Bermingham E, Lamb T, Neigel JE, Reeb CA, Saunders NC. Intraspecific Phylogeography: The Mitochondrial and Bridge between Population Genetics and Systematics. Annual Review of Ecology and Systematics. 1987; 18:489–522.

[37] Brower AV. Rapid morphological radiation and convergence among races of the butterfly *Heliconius erato* inferred from patterns of mitochondrial and evolution. Proceedings of the National Academy of Sciences of the United States of America. 1994; 91: 6491–6495. 8022810 10.1073/pnas.91.14.6491 PMC44228

[38] Moritz C, Dowling TE, Brown WM. Evolution of animal mitochondrial DNA: Relevance for population biology and systematics. Annu. Rev. Ecol. Syst. 1987; 18:269–92.

[39] Wilson AC, Cann RL, Carr SM, George M, Gyllensten UB, Helm-Bychowski KM, Higuchi RG, Palumbi SR, Prager EM, Sage RD, Stoneking M. Mitochondrial and two perspectives on evolutionary genetics. Biological Journal of the Linnean Society. 1985; 26, 375–400.

[40] Hebert PDN, Cywinska A, Ball SL, de Waard JR. Biological identifications through DNA barcodes. Proceedings of Royal Society of london. Series B: Biological Sciences. 2003; 270 (1512): 313–321. 10.1098/rspb.2002.2218 PMC1691236 12614582

[41] Ferreira ME, Grattapaglia D. Introdução ao uso de marcadores moleculares em análises genéticas. Brasilia: EMBRAPA, CENARGEM; 1998.

[42] Goldstein D, Schlötterer C. Microsatellites: evolution and applications. Oxford: Oxford University Press; 1999.

[43] Toth G, Gasdpari Z, Jurka J. Microsatellites in Different Eukaryotic Genomes: Survey and Analysis. Genome Research. 2000; 10: 967–981. 10899146 10.1101/gr.10.7.967 PMC310925

[44] Lopes DA, Cantagalli LB, Stuchi ALPB, Mangolin CA, Ruvolo-Takasusuki MCC. Population genetics of the sugarcane borer *Diatraea saccharalis* (Fabr.) (Lepidoptera: Crambidae). Acta Scientiarum. 2014; 36: 189–194.

[45] Silva-Brandão KL, Santos TV, Cônsoli FL, Omoto C. Genetic Diversity and Structure of Brazilian Populations of *Diatraea saccharalis* (Lepidoptera: Crambidae): Implications for Pest Management. Journal of Economic Entomology. 2015; 108(1):307–316. doi: 10.1093/jee/tou040 26470135 10.1093/jee/tou040

[46] Joyce AL, White WH, Nuessly GS, Solis MA, Scheffer SJ, et al Geographic Population Structure of the Sugarcane Borer, *Diatraea saccharalis* (F.) (Lepidoptera: Crambidae), in the Southern United States. Plos One, California. 2014; 9: 1–10. 10.1371/journal.pone.0110036 PMC4206286 25337705

[47] Joyce AL, Chicas MS, Cervantes LS, Paniagua M, Scheffer SJ, Solis AM. Host-plant associated genetic divergence of two *Diatraea* spp. (Lepidoptera: Crambidae) stemborers on novel crop plants. Ecology and Evolution. 2016; 1–13. 10.1002/ece3.2541 PMC5167014 28031813

[48] Walker F. List of the specimens of lepidopterous insects in the collection of the British Museum, part XXVII, Crambites and Tortricites, London; 1863.

[49] Robinson GS. The preparation of slides of Lepidoptera genitalia with special reference to the Microlepidoptera. Entomologist’s Gazette. 1976; 27:127–132.

[50] Doyle J.J.; Doyle J.L. Isolation of plant DNA from fresh tissue. Focus, Rockville, v. 12, p. 13–15, 1990.

[51] Folmer O, Black M, Hoeh W, Lutz R, Vrijenhoek R. DNA primers for amplification of mitochondrial cytochrome c oxidase subunit I from diverse metazoan invertebrates. Mol Mar Biol Biotechnol. 1994; 3: 294–299. 7881515

[52] Thompson J.D., Gibson T.J., Plewniak F., Jeanmougin F., and Higgins D.G. 1997 The CLUSTAL_Xwindows interface: Flexible strategies for multiple sequence alignment aided by quality analysis tools. Nucleic Acids Res. 25:4876–4882 9396791 10.1093/nar/25.24.4876 PMC147148

[53] Librado P. and Rozas J. DnaSP v5: A software for comprehensive analysis of DNA polymorphism data. Bioinformatics. 2009; 25: 1451–1452. doi: 10.1093/bioinformatics/btp187 19346325 10.1093/bioinformatics/btp187

[54] Excoffier L. and Lischer H.E. L.. Arlequin suite ver 3.5: A new series of programs to perform population genetics analyses under Linux and Windows. Molecular Ecology Resources. 2010; 10: 564–567. doi: 10.1111/j.1755-0998.2010.02847.x 21565059 10.1111/j.1755-0998.2010.02847.x

[55] Saitou N, Nei M. The neighbor-joining method: a new method for reconstructing phylogenetic trees. Mol Biol Evol. 1987; 4(4): 406–425. 3447015 10.1093/oxfordjournals.molbev.a040454

[56] Tamura K, Dudley J, Nei M, Kumar S. MEGA4: Molecular Evolutionary Genetics Analysis (MEGA) software version 4.0 Mol Biol Evol. 2007; 24(8):1596–9. doi: 10.1093/molbev/msm092 17488738 10.1093/molbev/msm092

[57] Pavinato VAC, Silva-Brandão KL, Monteiro M, Zucchi MI, Pinheiro JB, Dias FLF, Omoto C. Development and characterization of microsatellite loci for genetic studies of the sugarcane borer, *Diatraea saccharalis* (Lepidoptera: Crambidae). Genetics Molecular Research. 2013; 12:1631–1635. doi: 10.4238/2013.May.14.3 23765969 10.4238/2013.May.14.3

[58] Jerome Goudet and Thibaut Jombart. hierfstat: Estimation and Tests of Hierarchical F-Statistics. R package version 0.04–22. 2015. https://CRAN.R-project.org/package=hierfstat.

[59] Nei M. Estimation of average heterozygosity and genetic distance from a small number of individuals. Genetics. 1978; 89: 583–590. 17248844 10.1093/genetics/89.3.583 PMC1213855

[60] Kamvar ZN, Tabima JF, Grünwald NJ. Poppr: an R package for genetic analysis of populations with clonal, partially clonal, and/or sexual reproductionially clonal, and/or sexual reproduction. PeerJ. 2014; 2:e281 doi: 10.7717/peerj.281 24688859 10.7717/peerj.281 PMC3961149

[61] Jombart T. adegenet: a R package for the multivariate analysis of genetic markers. Bioinformatics. 2008; 24: 1403–1405. doi: 10.1093/bioinformatics/btn129 18397895 10.1093/bioinformatics/btn129

[62] Goyes, P. C. Caracterizacion morfologica y molecular de especies de Diatraea spp. (Lepidoptera: Crambidae). Instituto Colombiano para el Desarrollo de la Ciencia y la Tecnologia “Francisco José de Caldas”. 2008.

[63] Harrison RG. Animal mitochondrial DNA as a genetic marker in population and evolutionary biology. Trends in Ecol. Evol. 1989; 4:6–11. 10.1016/0169-5347(89)90006-2 21227301

[64] Jukes TH & Cantor CR (1969) Evolution of protein molecules In Munro HN, editor, Mammalian Protein Metabolism, pp. 21–132, Academic Press, New York.

[65] Avise JC. Phylogeography: the history and formation of species. Cambridge, UK: Harvard University Press 2000; 447 p.

[66] Risco, SH, Ferreira CE, Mendonça AF, Brandão JM, Sobral SM, Souza HD. Observaciones em Relacion a la Distribucion populacional de Diatraea spp. en la Region Cañavelera Del Nordeste de Brasil: Relatório Técnico do Programa Nacional de Melhoramento da Cana-de-açúcar (PLANALSUCAR), Maceió, Alagoas; 1975.

[67] Tajima F. The effect of change in population size on DNA polymorphism. Genetics. 1989; 123:597–601. 2599369 10.1093/genetics/123.3.597 PMC1203832

[68] Myers JG. The original habitat and hosts of three major sugarcane pests of tropical America (Diatraea, Castnia and Tomaspis). Imperial Institute of Entomology and Imperial College of Tropical Agriculture. 1932.

[69] Myers JG. The ecological distribution of some south american grass and sugar-cane borers (Diatraea spp. Lep. Pyralidae). Imperial Institute of Entomology and Imperial College of Tropical Agriculture. 1935.

[70] Patwardhan A, Ray S, Roy A. Molecular Markers in Phylogenetic Studies—A Review. J Phylogen Evolution Biol. 2014; 2: 131.

[71] Box HE. The more important insect pest of sugar cane in northern Venezuela. Proc. Hawaii Ent. Soc. 14:41–49. 1950

[72] Holloway TE, Haley WE, Loftin UC, Heinrich C. The sugar-cane borer in the United States. USDA Technical Bulletin. 1928; 41 77 pp.

[73] Elias-Gutierrez M, Jeronimo FM, Ivanova NV, Valdez-Moreno M, Hebert PDN. DNA barcodes for Cladocera and Copepoda from Mexico and Guatemala. Highlights and New Discoveries. Zootaxa 2008; 1–42.

[74] Rock J, Costa FO, Walker DD, North AW, Hutchinson WF, Carvalho GR. DNA barcodes of fish of the Scotia Sea, Antarctica indicate priority groups for taxonomic and systematics focus. Antarctic Science. 2008; 20: 253–22.

[75] Shere-Kharwar A, Magdum S. Assessment Of Different Body Parts For Serving As Most Preferred Tissue For DNA Isolation In Moths (Lepidoptera: Sphingidae). Life Science Bulletin. 2012; (1):123–125.

[76] Shere-Kharwar A, Magdum S, Khedkar GD, Gupta S, Zambare V. Moth Legs: Excellent Source Of Tissue For Dna Extraction (Lepidoptera:Noctuidae). Indian J.L.Sci. 2013; 2: 35–37

[77] Elton CS. The ecology of invasions by animals and plants. Methuen, London 1958.

[78] Debach P, Rosen D. Biological control by natural enemies. Cambridge University Press, Cambridge 1991.

[79] Nei M. Molecular Population Genetics and Evolution. North-Holland, Oxford 1975. 1228006

[80] Templeton AR. The theory of speciation via the founder principle. Genetics. 1980; 94:11–20. 10.1093/genetics/94.4.1011 PMC1214177 6777243

[81] Barton NH, Charlesworth B. Genetic revolutions, founder effects and speciation. Annu Rev Ecol Syst. 1984; 15:133–164

[82] Hartl DL, Clark AG. Principles of Population Genetics. Sinauer Associates Inc. Publishers, USA; 2007.

[83] Brower AVZ, Boyce TM. Mitochondrial DNA variation in monarch butterflies. Evolution 1991; 45: 1281–1286. doi: 10.1111/j.1558-5646.1991.tb04393.x 28564167 10.1111/j.1558-5646.1991.tb04393.x

[84] Chapco W, Kelln RA, McFadyen DA. Intraspecific mitochondrial DNA variation in the migratory grasshopper, Melanoplus sanguinipes. Heredity. 1992; 69: 547–557.

[85] Zehnder GW, Sandall L, Tisler AM, Powers TO. Mitochondrial DNA diversity among 17 geographic populations of *Leptiotarsa decemlineata* (Coleoptera: Chrysomelidae). Ann Entomol Soc Am. 1992; 85:234–240.

[86] Bogdanowicz SM, Wallner WE, Bell J, O’ Dell TM, Harrison RG. Asian gypsy moths (Lepidoptera, Lymantriidae) in North America—evidence from molecular data. Ann Entomol Soc Am. 1993; 86:710–715

[87] Martin A, Simon C. Differing levels of among-population divergence in the mitochondrial DNA of periodical cicadas related to historical biogeography. Evolution. 1990; 44: 1066–1080. doi: 10.1111/j.1558-5646.1990.tb03826.x 28569016 10.1111/j.1558-5646.1990.tb03826.x

[88] Pashley DP. Host-associated differentiation in army-worms (Lepidoptera: Noctuidae): An allozymic and mitochondrial DNA perspective In: Loxdale HD, Hollander JD Electrophoretic Studies on Agricultural Pests 103–114 Clarendon, Oxford 1989.

[89] Lipscomb D, Platnick N, Wheeler Q. The intellectual content of taxonomy: A comment on DNA taxonomy. Trends Ecol. Evol. 2003; 18:65–68.

[90] Sneath Pha R. R. Sokal RR. Numerical taxonomy. W. H. Freeman and Company, San Francisco; 1973.

[91] Nei M. Estimation of average heterozygosity and genetic distance from a small number of individuals.Genetics. 1978; 89(3):583–590. 17248844 10.1093/genetics/89.3.583 PMC1213855

[92] Gepts P. Who owns biodiversity and how should the owners be compensated? Plant Physiol. 2004; 134: 1295–1307. doi: 10.1104/pp.103.038885 15084724 10.1104/pp.103.038885 PMC419806

[93] Tischendorf L, Bender DJ, Fahrig L. Evaluation of patch isolation metrics in mosaic landscapes for specialist vs. generalist dispersers. Landscape Ecol. 2003; 18: 41–50.

[94] Vialatte A, Dedryver CA, Simon J-C, Galman M, Plantegenest M. Limited genetic exchanges between populations of an insect pest living on uncultivated and related cultivated host plants. Proc R Soc B. 2005; 1567:1075–1082. 10.1098/rspb.2004.3033 PMC1599878 16024367

[95] Stireman JO, Nason JD, Heard SB. Host-associated genetic differentiation in phytophagous insects: General phenomenon or isolated exceptions? Evidence from a goldenrod-insect community. Evolution. 2005; 59: 2573–2587. 16526505

[96] Simpson GG. Tempo and mode in evolution. Columbia University Press, New York; 1949.

[97] Simpson GG. The major features of evolution. Columbia University Press, New York; 1953.

[98] Mitter C, Farrell B, Futuyma DJ. Phylogenetic studies of insect-plant interactions: insights into the genesis of diversity. Trends Ecol. Evol. 1991; 6:290–293. doi: 10.1016/0169-5347(91)90007-K 21232484 10.1016/0169-5347(91)90007-K

[99] Schluter D. The ecology of adaptive radiation. Oxford University Press, Oxford; 2000.

[100] Yoder JB, Clancey E, Des Roches S, Eastman JM, Gentry L, Godsoe W. Ecological opportunity and the origin of adaptive radiations. J. Evol. Biol. 2010; 23:1581–596. doi: 10.1111/j.1420-9101.2010.02029.x 20561138 10.1111/j.1420-9101.2010.02029.x

[101] Peterson MA, Denno RF. Life history strategies and the genetic structure of phytophagous insect populations In: Mopper S. and Strauss S. (eds) Genetic Structure and Local Adaptation in Natural Insect Populations, pp. 263±322. Chapman & Hall, New York; 1998.

[102] Hayward KJ. A broca da cana-de-açúcar. Brasil Açucareiro, Rio de Janeiro. 1943; 11: 69–74.

[103] Caixeta DF. Dispersão de machos de Diatraea saccharalis (Fabricius) (Leipdoptera: Crambidae) em cana-de-açúcar. Dissertação (mestrado)—Universidade Estadual Paulista, Faculdade de Ciências Agrárias e Veterinárias. 2010.

[104] Pavinato VAC. Variabilidade genética, estruturação populacional e busca de variação alélica em locos associados à adaptação inseto-planta em Diatraea saccharalis (Fabr. 1794) (Lepidoptera: Crambidae). 149 p. Tese (Doutorado em Genética e Biologia Molecular)–Instituto de Biologia, Universidade Estadual de Campinas, Campinas, 2014.

[105] Nascimento JB. Diversidade genética estrutura populacional de Diatraea saccharalis (Fabricius) (Lepidoptera: Crambidae) nas culturas do arroz (Oryza sativa L.) e cana-de-açúcar (Saccharum officinarum L.). Universidade Federal de Goiás, Escola de Agronomia (EA), Programa de Pós-Graduação em Agronomia, Goiânia, 2015.

