# Supplementary material for: Morphological and molecular characterization of Brazilian populations of Diatraea saccharalis (Fabricius, 1794) (Lepidoptera: Crambidae) and the evolutionary relationship among species of Diatraea Guilding
Source: PLoS One. 2017 Nov 16;12(11):e0186266. doi: 10.1371/journal.pone.0186266 (PMC5690654; doi:10.1371/journal.pone.0186266)
Supplement: S1 Table — F = forward primer sequences; R = reverse primer sequences; Ta = annealing temperature. (PDF) [file pone.0186266.s001.pdf]

Supplementary Table 1. Characteristics of the 11 microsatellite loci from *Diatraea saccharalis*.

Locus	GeneBank accession	Primer nucleotide sequence (5'-3')	Repeat motif	Ta (°C)	Size range (bp)
Dsc1	GF111048	F CGAGGCTATATTTGCGTGTG<br>R GATGATGGAGTTGGAAGGTGA	(TG)10	56	180-192
Dsc2	GF111061	F GCGGTGCCTCTTTGTCATA<br>R TTGACCAACTACTGCAAGACG	(CA)19	60	188-230
Dsc3	GF111049	F CCATCAAGCTCCTTCTAAGAGAC<br>R CCTTGCTCAGTTACCATTCG	(AC)11	56	250-274
Dsc5	GF111050	F TCTTGCCTTTGCTCTTGAAA<br>R GCGGGGTCAGCTAGTTATTC	(TG)19	60	146-190
Dsc7	GF111051	F TGTCGAGCTACTCCATGCTT<br>R TGAGACTGAACACTGGCAAGA	(ATG)6	60	214-250
Dsc9	GF111052	F AACCTTCGATGAGCTACTGC<br>R TGTGGTGATTTGTTTGCTTG	(TG)16	56	160-182
Dsc10	GF111060	F GGTCCGCGTTTGTTATTGTT<br>R TCAAGTGCTCCTTAAAACACGA	(GT)7	56	270-280
Dsc11	GF111990	F ATACGGCTTCATTCGCTTC<br>R GGTTTCGCACTCATCACG	(GT)10	54	220-228
Dsc13	GF111053	F CGTGGACTAACCCATAGAAGAT<br>R GGTTTAGCAGAACTTGGCATA	(GT)18	54	220-270
Dsc19	GF111058	F CACACACGAACACACACGA<br>R ATGGTTGGGTCTTTCCTTTT	(CA)10	60	160-170
Dsc20	GF111059	F TTGGCAGAGTTGTGGGTAAC<br>R ACAGCAGCATCATCAGAAGG	(AG)8	54	222-230

F = forward primer sequences; R = reverse primer sequences; Ta = annealing temperature;
