# Supplementary material for: Morphological and molecular characterization of Brazilian populations of Diatraea saccharalis (Fabricius, 1794) (Lepidoptera: Crambidae) and the evolutionary relationship among species of Diatraea Guilding
Source: PLoS One. 2017 Nov 16;12(11):e0186266. doi: 10.1371/journal.pone.0186266 (PMC5690654; doi:10.1371/journal.pone.0186266)
Supplement: S2 Table — (PDF) [file pone.0186266.s002.pdf]

Supplementary Table 2 – Hierarchical analysis of molecular variance (AMOVA) for population genetic structure of *Diatraea saccharalis* with a mithocondrial (COI) region marker.

Hierarchical levels	d.f.	Sum of Squares	Variance components	Variance (%)	Fixation Indices	P valor
Two-hierarchical-levels						
Among Populations	2	0.184	-0.00067	-0.63	-0.00625	0.45974
Within Populations	79	8.463	0.10712	100.63		
Total	81	8.646	0.10645			
Two-hierarchical-levels						
Among Host	1	0.076	-0.00079	-0.74	-0.00742	0.6998
Within Host	80	8.57	0.10712	100.74		
Total	81	8.646	0.10634			
Three-hierarchical-levels						
Among Populations	2	0.184	0.00271	2.54	F_{SC} = -0.046	1
Among host within populations	2	0.088	-0.00478	-4.48	F_{CT} = 0.02537	0.3993
Within Host	77	8.375	0.10877	101.94	F_{ST} = -0.0194	0.7271
Total	81	8.646	0.10669			
