# Supplementary material for: Morphological and molecular characterization of Brazilian populations of Diatraea saccharalis (Fabricius, 1794) (Lepidoptera: Crambidae) and the evolutionary relationship among species of Diatraea Guilding
Source: PLoS One. 2017 Nov 16;12(11):e0186266. doi: 10.1371/journal.pone.0186266 (PMC5690654; doi:10.1371/journal.pone.0186266)
Supplement: S3 Table — (PDF) [file pone.0186266.s003.pdf]

Supplementary Table 3. Estimates of pairwise Fst (lower diagonal) and p-values (upper diagonal) among populations of *Diatraea saccharalis* and *Diatraea impersonatella*, based on the variation of the mitochondrial COI gene.

	Jaboticabal_Sugarcane	Morrinhos_Corn	Morrinhos_Sugarcane	Piracicaba_Corn	Piracicaba_Sugarcane	Maceio_Sugarcane
Jaboticabal_Sugarcane		0.436	1.000	1.000	1.000	0.000
Morrinhos_Corn	**0.05546**		1.000	0.448	0.300	0.000
Morrinhos_Sugarcane	**-0.33172**	**-0.31765**		1.000	1.000	0.008
Piracicaba_Corn	**-0.01366**	**-0.00878**	**-0.31555**		1.000	0.000
Piracicaba_Sugarcane	**-0.02813**	**0.04**	**-0.29095**	**-0.02391**		0
Maceio_Sugarcane	**0.98657**	**0.97568**	**0.96682**	**0.9829**	**0.9857**	
