# Supplementary material for: Morphological and molecular characterization of Brazilian populations of Diatraea saccharalis (Fabricius, 1794) (Lepidoptera: Crambidae) and the evolutionary relationship among species of Diatraea Guilding
Source: PLoS One. 2017 Nov 16;12(11):e0186266. doi: 10.1371/journal.pone.0186266 (PMC5690654; doi:10.1371/journal.pone.0186266)
Supplement: S4 Table — (PDF) [file pone.0186266.s004.pdf]

Supplementary Table 4. Genetic variability and coefficient of inbreeding for microsatellite loci evaluated in *Diatraea saccharalis* populations.

**Locus**	**A**	**Ho ± SE**	**He ± SE**	**F_{IS} ± SE**
Dsc1	3	0.3125 ± 0.027	0.54775 ± 0.036	0.42275 ± 0.060
Dsc2	3	0.60025 ± 0.078	0.50575 ± 0.051	-0.20625 ± 0.161
Dsc3	6	0.3645 ± 0.104	0.59025 ± 0.077	0.3675 ± 0.184
Dsc5	4	0.3915 ± 0.101	0.632 ± 0.005	0.37825 ± 0.162
Dsc7	4	0.47925 ± 0.100	0.504 ± 0.026	0.01375 ± 0.245
Dsc9	6	0.28575 ± 0.121	0.51675 ± 0.109	0.472 ± 0.156
Dsc10	3	0.34975 ± 0.110	0.467 ± 0.095	0.34225 ± 0.152
Dsc11	7	0.27 ± 0.160	0.22675 ± 0.134	-0.1885 ± 0.013
Dsc13	6	0.602 ± 0.088	0.6325 ± 0.081	0.05225 ± 0.056
Dsc19	7	0.88525 ± 0.057	0.625 ± 0.026	-0.4135 ± 0.045
Dsc20	2	0.084 ± 0.068	0.12275 ± 0.071	0.3455 ± 0.309
**Average**	**4.64**	**0.42**	**0.49**	**0.14**

A - Number of alleles, Ho - Observed heterozygosity, He - Expected heterozygosity, F_{IS} - Inbreeding coefficient
