# Supplementary material for: Morphological and molecular characterization of Brazilian populations of Diatraea saccharalis (Fabricius, 1794) (Lepidoptera: Crambidae) and the evolutionary relationship among species of Diatraea Guilding
Source: PLoS One. 2017 Nov 16;12(11):e0186266. doi: 10.1371/journal.pone.0186266 (PMC5690654; doi:10.1371/journal.pone.0186266)
Supplement: S5 Table — (PDF) [file pone.0186266.s005.pdf]

Supplementary Table 5 – Nuclear microsatellite private alleles observed in four populations of *Diatraea saccharalis.*

Population	Locus	Allele	Frequency
Jaboticabal_Sugarcane	Dsc11	210	0.1363
	Dsc11	254	0.0227
	Dsc11	256	0.0454
Morrinhos_Corn	Dsc3	282	0.4375
	Dsc9	188	0.1428
	Dsc11	194	0.1250
	Dsc11	220	0.0625
	Dsc13	314	0.1875
	Dsc19	203	0.1250
Piracicaba_Sugarcane	Dsc2	228	0.2391
	Dsc19	169	0.0208
	Dsc19	175	0.0416
