# Supplementary material for: Morphological and molecular characterization of Brazilian populations of Diatraea saccharalis (Fabricius, 1794) (Lepidoptera: Crambidae) and the evolutionary relationship among species of Diatraea Guilding
Source: PLoS One. 2017 Nov 16;12(11):e0186266. doi: 10.1371/journal.pone.0186266 (PMC5690654; doi:10.1371/journal.pone.0186266)
Supplement: S6 Table — (PDF) [file pone.0186266.s006.pdf]

Supplementary Table 6 - Estimates of pairwise Fst among the *Diatraea saccharalis* populations based on the variation of eleven microsatellite loci.

	Jaboticabal_Sugarcane	Morrinhos_Corn	Piracicaba_Sugarcane	Piracicaba_Corn
Jaboticabal_Sugarcane	0			
Morrinhos_Corn	0.1415	0		
Piracicaba_Sugarcane	0.1578	0.1046	0	
Piracicaba_Corn	0.1812	0.0835	0.1533	0
